# Burden of disease of respiratory syncytial virus in older adults and adults considered at high risk of severe infection

**DOI:** 10.14745/ccdr.v51i01a04

**Published:** 2025-01-02

**Authors:** Elissa M Abrams, Pamela Doyon-Plourde, Phaedra Davis, Liza Lee, Abbas Rahal, Nicholas Brousseau, Winnie Siu, April Killikelly

**Affiliations:** 1 Public Health Agency of Canada, Ottawa, ON; 2 Section of Allergy and Clinical Immunology, Department of Pediatrics, University of Manitoba, Winnipeg, MB; 3 Division of Allergy and Immunology, Department of Pediatrics, University of British Columbia, Vancouver, BC; 4 School of Epidemiology and Public Health, University of Ottawa, Ottawa, ON; 5 Institut national de la santé publique du Québec, Québec, QC

**Keywords:** respiratory syncytial virus, adults, burden of disease, surveillance, epidemiology

## Abstract

**Background:**

Availability of new vaccines for adults has increased interest in understanding Canada’s respiratory syncytial virus (RSV) burden in older adults and adults considered at high risk of severe infection.

**Objective:**

To characterize the burden of RSV disease in Canada by joint analysis of the published literature and hospitalization data from a healthcare administrative database.

**Methods:**

Electronic databases of published literature were searched to identify studies and systematic reviews reporting data on outpatient visits, hospitalizations, intensive care unit (ICU) admissions and deaths associated with RSV infection in adults. For the hospitalization data analysis, hospital discharge records were extracted from the Canadian Institute of Health Information Discharge Abstract Database for all patients admitted to an acute care facility for RSV infection defined by ICD-10 codes from 2010 to 2020 and 2021 to 2023.

**Results:**

Overall, 26 studies, including seven systematic reviews, were identified and summarized. Evidence suggests that medically attended RSV respiratory tract infections (RTI) are frequently causing 4.7%–7.8% of symptomatic RTI in adults 60 years of age and older. Incidence of RSV RTI increases with age and presence of underlying medical conditions. This trend was consistently observed across all RSV clinical outcomes of interest. Patients who reside in long-term care or other chronic care facilities have a higher likelihood of severe clinical outcomes compared to patients with other living situations upon hospital admission. Approximately 10% of older adults hospitalized with RSV infection require ICU admission. Although data are limited, the case fatality ratio (CFR) among those admitted to hospital varies between 5% and 10%. Some evidence suggests that RSV burden may be close to the influenza burden in older adults. In general, the results from the Canadian hospitalization data support the rapid review findings. Rates of hospitalization, ICU admission and death associated with RSV all increased with age, with 16% of hospitalizations resulting in ICU admission and with an in-hospital CFR of 9%.

**Conclusion:**

In adults, the burden of severe RSV outcomes in general increases with age and presence of comorbidities.

## Introduction

Respiratory syncytial virus (RSV) is commonly recognized as a significant respiratory pathogen mostly affecting young children under 24 months of age and older adults. Although the burden of disease in the older adult demographic can be substantial, with older adults experiencing more severe disease compared to younger populations, this is not as well described as it is in children and for other pathogens such as influenza. It has been estimated that globally, RSV is associated with approximately 336,000 hospitalizations and 14,000 in-hospital deaths each year in adults 65 years and older ((1)). Additionally, evidence suggests that younger adults living with underlying medical conditions, such as immunocompromising conditions and chronic cardiopulmonary disease, are at high risk of severe RSV infection and complications ((2,3)). Nonetheless, RSV remains generally underrecognized as a cause of severe respiratory tract infection (RTI) in adults.

The RSV vaccine landscape has evolved dramatically in the past year. While previously there were no vaccine products available for adults, there are currently three RSV vaccines being considered in Canada. As of February 2024, The GSK RSVPreF^3^ vaccine (Arexvy) and the Pfizer RSVpreF vaccine (Abrysvo) are approved by Health Canada for adults 60 years of age and older and the Moderna mRNA-1345 RSV vaccine is under review. As vaccination will be available to older adults for the first time, there is a need for a more nuanced understanding of the burden of RSV disease to inform risk and age-based vaccine recommendations especially in a Canadian context although comments on policy were out of scope for this document. Therefore, this rapid review aimed to evaluate RSV burden of disease in adults from high-income countries (Canada, United States, European countries, Australia). Additionally, hospitalization data from the Canadian Institute for Health Information (CIHI) Discharge Abstract Database (DAD) were analyzed to further describe RSV burden in Canada. This report compiles evidence derived from the literature and the Canadian discharge database in order to present a comprehensive picture of the RSV burden of disease to inform immunization guidance development in adults.

## Methods

### Rapid review

**Search strategies:** The search strategy was developed by a research librarian from Health Canada and the Public Health Agency of Canada. OVID Embase, MEDLINE, Global Health and ProQuest Public Health databases were searched from 1995 to November 2022, and again on September 1, 2023, to identify recent studies evaluating RSV burden of disease in adults (**Appendix**, Supplemental material S1–S6). Canadian respiratory virus surveillance experts were also contacted for any additional data. After removal of duplicates, references were uploaded in DistillerSR online software (Evidence Partners Inc., Ottawa, Ontario).

**Study selection:** Two reviewers screened titles and abstracts for study eligibility. Full texts of selected studies were then evaluated. A third independent reviewer assessed citations marked for exclusion, with disagreements resolved through discussion. Reference lists of included studies were also screened for relevant articles on RSV burden in high-income countries.

**Eligibility criteria:** Inclusion was limited to studies reporting data on RSV infection in adults, with a focus on adults 50 years of age and older and individuals 18 years of age and older with underlying medical conditions. The evaluation of RSV burden of disease focused on clinical outcomes of interest including medically attended RSV RTI, hospitalizations, intensive care unit (ICU) admissions, and death associated with RSV infection (Supplemental material S7). Observational studies, randomized controlled trials (RCTs) and systematic reviews (SRs) were included. Exclusion criteria were populations of other ages, and studies that did not report on outcomes of interest. The focus was on high-income countries, although studies from low- and middle-income countries were included.

**Data extraction and data synthesis:** One reviewer extracted data from each article, verified by a second reviewer. Disagreements were resolved through discussion. Data extracted included study design, study period, population characteristics, outcome definitions, sample size, number of events and effect measures. When reported in included studies, results comparing RSV and influenza burden of disease were extracted. Results were synthesized narratively based on the study population and outcomes. Subgroups of interest included long-term care (LTC) residents, adults with immunocompromising conditions and adults with chronic medical conditions.

### Canadian hospitalization data

**Data sources:** Hospital discharge records were extracted from the CIHI DAD which contains data from acute care facilities from all provinces and territories, except Québec, representing 78% of the Canadian population ((4)). Population demographic data (i.e., age group) were obtained from the Statistics Canada website ((5)).

Respiratory syncytial virus hospitalizations were identified using the International Classification of Disease, Tenth Revision (ICD-10) codes J12.1, J20.5, J21.0 or B97.4. Respiratory syncytial virus hospitalizations were classified as one of the aforementioned ICD-10 codes recorded as anywhere from diagnosis 1 through 25. Hospitalizations were further stratified to determine the hospitalizations due to RSV, which was defined as one of the aforementioned ICD-10 codes recorded as diagnosis 1.

Results were presented in two groups: hospitalizations due to RSV and hospitalizations associated with RSV. Hospitalizations due to RSV provides data on direct burden of RSV in the hospitalized population as it includes only patients where RSV was coded as the most responsible diagnosis or condition for the patient’s stay in a facility (RSV-related ICD-10 code as diagnosis 1). Hospitalizations associated with RSV provide a general sense of the prevalence of RSV in the hospitalized population as it includes both patients where RSV was identified as the condition considered the most responsible for the stay in a facility and where it was diagnosed or present in the patient during their stay in the facility (RSV-related ICD-10 code found anywhere from diagnosis 1 through 25).

### Analytic cohort

All patients admitted to an acute care facility with RSV between September 2010 to August 2020 and September 2021 to August 2023 (12 respiratory virus seasons spanning September through August of the following year) were included in the analysis. Due to public health measures enacted for the COVID-19 pandemic, there was almost no RSV activity in the 2020–2021 season ((6)); therefore, this season was excluded from the analysis as it did not reflect normal seasonal activity.

Data on ICU admissions and in-hospital deaths were also extracted. Diagnosis codes were not available specifically for ICU admissions and deaths; therefore, their classifications (associated with or due to RSV) were based on whether the initial hospitalization was associated with or due to RSV.

Risk factors of interest were also determined by diagnosis information based on ICD-10 classification codes and were chosen based on prior known associations with severe RSV outcomes. Diagnosis codes were considered mutually exclusive (i.e., one individual hospitalized for RSV with multiple risk factors of interest were counted in each individual risk factor category). All diagnoses and conditions that are present on a patient’s record from diagnosis 1 through 25 were included in determining their risk factors. Risk factors of interest for the Canadian hospitalization data analysis included RTI, chronic obstructive pulmonary disease (COPD), immunocompromising conditions, cardiovascular disease, diabetes and chronic kidney disease. The list of ICD-10 codes used to define a risk factor is found in Supplemental material S8.

**Data synthesis:** The number of hospitalizations, ICU admissions and in-hospital death analyses were both aggregated and stratified by season and age groups where appropriate. Hospitalizations were also presented as rates aggregated by season and stratified by age groups (50–59 years, 60–69 years, 70–79 years and ≥80 years). Moreover, ICU admission rates and case fatality ratios (CFR) were presented by age group aggregated across the study period. The population of all provinces and territories, except Québec, by age groups was used to calculate rates per 100,000 population. The 18–49 years age group was included in the analysis for risk factors of interest. Data on risk factors were aggregated across age groups and seasons. Descriptive data analyses were performed in SAS 9.4 and figures were produced using Microsoft Excel. The results from the analysis of Canadian hospitalization data were compared with the evidence from the rapid review. Results from both sources are presented and summarized by outcome of interest, except for medically attended RSV RTI for which data was only available from the rapid review.

## Results

**Figure 1:**
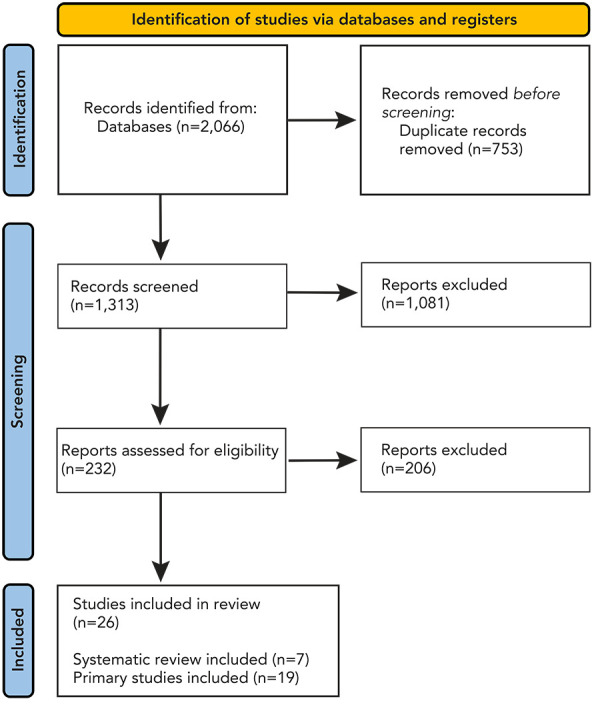
Study selection PRISMA flow diagram Abbreviation: PRISMA, Preferred Reporting Items for Systematic reviews and Meta-Analyses

After deduplication, 1,313 references were screened for study eligibility in the rapid review (**Figure 1**). Overall, 26 articles, including 7 SRs, were incorporated into the narrative synthesis of RSV burden of disease in adults (Supplemental material S9).

Between September 2010 to August 2020 and September 2021 to August 2023, there were a total of 19,436 recorded hospitalizations associated with RSV among adults 50 years of age and older, of which 6,314 were due to RSV (**Table 1**).

**Table 1:** Total hospitalizations, intensive care unit admissions and in-hospital deaths associated with and due to respiratory syncytial virus, adults aged 50 years of age and older, seasons 2010–2011 to 2019–2020 and 2021–2022 to 2022–2023^a,b^

Season	Hospitalizations		Rate of hospitalizations per 100,000 population		ICU admissions		In-hospital deaths	
	Associated with RSV^c^	Due to RSV^d^	Associated with RSV^c^	Due to RSV^d^	Associated with RSV^c^	Due to RSV^d^	Associated with RSV^c^	Due to RSV^d^
2010–2011	238	90	3	1	51	13	28	11
2011–2012	179	53	2	1	41	4	19	1
2012–2013	591	211	6	2	113	30	49	13
2013–2014	663	251	7	3	149	38	59	20
2014–2015	1,342	402	14	4	247	42	120	20
2015–2016	921	317	9	3	177	43	77	23
2016–2017	2,225	695	22	7	393	85	190	49
2017–2018	2,338	706	22	7	374	69	205	45
2018–2019	2,891	928	27	9	482	90	232	52
2019–2020	2,213	753	20	7	333	70	193	48
2021–2022	1,330	469	12	4	188	40	118	34
2022–2023	4,505	1,439	39	13	636	118	378	81
Total	19,436	6,314	-	-	3,184	642	1,668	397
Average/season	1,620	526	16	5	265	54	139	33

Abbreviations: ICU, intensive care unit; RSV, respiratory syncytial virus; -, not applicable

^a^ Canada, excluding Québec

^b^ Canadian Discharge Abstract Database

^c^ Hospitalizations associated with RSV were identified using ICD-10 codes J12.1, J20.5, J21.0 or B97.4, found anywhere from diagnosis 1 through 25

^d^ Hospitalizations due to RSV were identified using ICD-10 codes J12.1, J20.5, J21.0 or B97.4, recorded as diagnosis 1

### Medically attended respiratory syncytial virus respiratory tract infection

**Rapid review:** Seven SRs and six observational studies describing the incidence of medically attended RSV RTI in older adults as well as adults with underlying medical conditions were identified; some included Canadian data (n=3), but none were restricted to Canada (Supplemental material S9). Some specific findings are specified here; full details of all studies are included in Supplemental material S9. In adults 60 years of age and older, a SR of developed countries, including Canada, found that RSV caused between 4.7% and 7.8% of symptomatic respiratory infections ((7)). Overall, the incidence of medically attended RSV RTI increased with age ((8,9)). For instance, a SR and meta-analysis (MA) found that rates of medically attended RSV RTI among adults from the United States (US) increased from 934 per 100,000 population in adults 18–49 years of age to 1,519 per 100,000 population in adults 65 years of age and older ((10)). Factors associated with severe RSV infection in adults 65 years of age and older included age and the presence of underlying medical conditions (i.e., cardiorespiratory disease, diabetes and immunocompromising conditions). In a prospective US cohort study of adults 60 years of age and older, incidence was almost two times higher among adults with chronic cardiopulmonary disease compared to those without (incidence rate ratio [IRR] of 1.89; 95% confidence interval [CI]: 1.44–2.48) ((11)). Although evidence was limited, studies suggest that RSV incidence is high in younger adults (i.e., 18–59 years) with certain medical conditions and is somewhat similar to adults 65 years of age and older. A cross-sectional study from the US of the annual incidence of medically attended RSV found that incidence was highest in adults 85 years of age and older, followed by adults 65 years of age and older, and then followed closely by adults 18–59 years of age considered at high risk of severe RSV including those with cardiorespiratory disease or immunocompromising conditions ((3)).

### Hospitalization associated with respiratory syncytial virus infection

**Rapid review:** Six SRs and 15 observational studies, including four Canadian studies, described the incidence of hospitalization associated with RSV infection. In general, studies found that the incidence increased consistently with age. For instance, a prospective Canadian population-based surveillance study found the following average seasonal RSV hospitalization incidence rates per 100,000 population between 2012 and 2015:13.9 (95% CI: 9.9–17.9) in adults aged 50–59 years, 43.7 (95% CI: 34.2–51.2) in adults aged 60–69 years, 88.6 (95% CI: 71.0–106.1) in adults aged 70–79 years and 282.5 (95% CI: 238.2–326.8) in adults 80 years of age and older ((2)). A SR found that depending on age and risk factors, adults 18 years of age and older with chronic medical conditions have higher rates of hospitalization associated with RSV compared to those without the condition ((10)). The authors reported rates ranging from 1.2–1.3 times higher for adults with obesity to 27.6 times higher for those 20–39 years of age with congestive heart failure (CHF) ((10)). Similarly, a retrospective cohort study from Ontario found that among adults 18 years of age and older who had a hospitalization associated with RSV between September 2010 and August 2017, 35.4% had CHF, 44.7% had COPD, 32.2% had asthma and 38.4% had immunocompromising conditions; in addition, hospitalizations associated with RSV increased from 2010–2011 to 2018–2019 ((12)). Another Canadian study found that of adults 50 years of age and older who had a hospitalization associated with RSV over the 2012–2015 seasons, almost all (98.1%) had at least one comorbidity with the most frequent being vascular (71.3%), cardiac (55.5%), pulmonary (48.2%), renal (48.2%) and endocrine (33.2%) conditions; 26.8% were immunocompromised ((2)).

**Figure 2:**
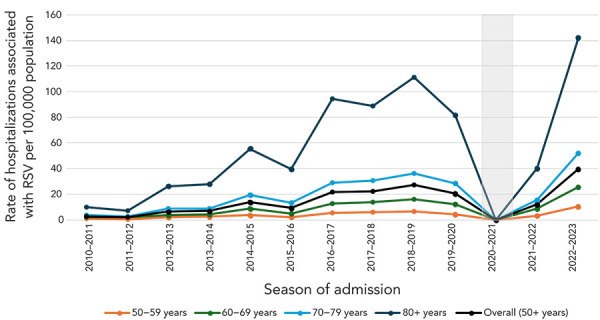
Rate of hospitalizations associated with respiratory syncytial virus, by age group (years), seasons 2010–2011 to 2019–2020 and 2021–2022 to 2022–2023^a,b,c,d^ Abbreviation: RSV, respiratory syncytial virus ^a^ Canada, excluding Québec ^b^ Canadian Discharge Abstract Database ^c^ The shaded area represents the 2020–2021 season where RSV hospitalizations were low due to public health measures enacted during the COVID-19 pandemic. The 2020–2021 season was excluded from other analyses ^d^ Hospitalizations associated with RSV were identified using ICD-10 codes J12.1, J20.5, J21.0 or B97.4, found anywhere from diagnosis 1 through 25

**Figure 3:**
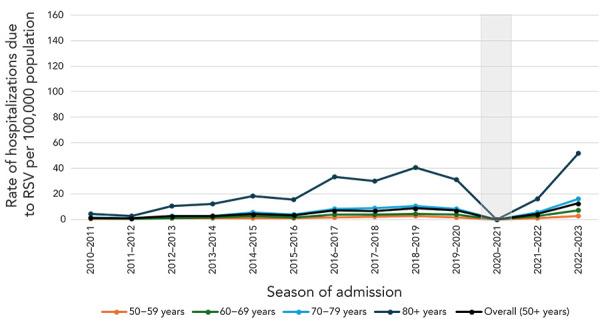
Rate of hospitalizations due to respiratory syncytial virus, by age group (years), seasons 2010–2011 to 2019–2020 and 2021–2022 to 2022–2023^a,b,c,d^ Abbreviation: RSV, respiratory syncytial virus ^a^ Canada, excluding Québec ^b^ Canadian Discharge Abstract Database ^c^ The shaded area represents the 2020–2021 season where RSV hospitalizations were low due to public health measures enacted during the COVID-19 pandemic. The 2020–2021 season was excluded from other analyses ^d^ Hospitalizations due to RSV were identified using ICD-10 codes J12.1, J20.5, J21.0 or B97.4, recorded as diagnosis 1

**Canadian hospitalization data:** Rates of hospitalizations associated with RSV among older adults in Canada were generally increasing between seasons 2011–2012 and 2017–2018 across all age groups until RSV activity was interrupted by the COVID-19 pandemic between seasons 2019–2020 to 2021–2022 (**Figure 2**). Overall, the average rate of hospitalization associated with RSV among adults 50 years of age and older was 16 per 100,000 population and the average rate of hospitalization associated with RSV per 100,000 population by age groups were the following: 4 in adults 50–59 years old, 10 in adults 60–69 years old, 22 in 70–79 years and 63 in adults 80 years of age and older. The rates of hospitalizations due to RSV among older adults in Canada followed the same trend; however, rates were much lower (**Figure 3**). Rates of hospitalization associated with and due to RSV increased with age ((6,13)).

A total of 21,258 hospitalizations associated with RSV among adults 18 years of age and older were reported across the 12 seasons (**Table 2**). Of these hospitalizations, 76.4% were reported to have at least one risk factor of interest, 34.6% were reported to have at least two of these risk factors and 9.1% were reported to have at least three of these risk factors. Among these 21,258 hospitalizations, 30.0% reported having COPD, 29.6% had diabetes, 23.4% had cardiovascular disease, 16.8% had an immunocompromising condition, 15.8% had a respiratory tract infection and 6.3% had chronic kidney disease.

**Table 2:** Number and percent hospitalizations associated with respiratory syncytial virus with a risk factor of interest, adults aged 18 years of age and older, seasons 2010–2011 to 2019–2020 and 2021–2022 to 2022–2023^a,b^

Risk factor of interest	Number of hospitalizations^c^	Percentage (%)^d^
Chronic obstructive pulmonary disease	6,360	30.0
Diabetes	6,276	29.6
Cardiovascular disease	4,965	23.4
Immunosuppressive conditions	3,564	16.8
Respiratory tract infection	3,344	15.8
Chronic kidney disease	1,336	6.3
Total number of hospitalizations associated with RSV^e^	21,258	-
**Total number of risk factors of interest**		
At least 1	16,250	76.4
2 or more	7,345	34.6
3 or more	1,928	9.1
4 or more	297	1.4

Abbreviations: RSV, respiratory syncytial virus; -, not applicable

^a^ Canada, excluding Québec

^b^ Canadian Discharge Abstract Database

^c^ Number of hospitalizations by risk factor will not equal 21,258 hospitalizations as one patient may have multiple risk factors and the occurrence of each risk factor was considered mutually exclusive

^d^ Percentage of hospitalizations will exceed 100% as one patient may have multiple risk factors and the occurrence of each risk factor was considered mutually exclusive

^e^ Hospitalizations associated with RSV were identified using ICD-10 codes J12.1, J20.5, J21.0 or B97.4, found anywhere from diagnosis 1 through 25

### Intensive care unit admission associated with respiratory syncytial virus infection

**Rapid review:** Two SRs and nine observational studies, including three Canadian studies, reported data on ICU admission associated with RSV infection. Full results are listed in Supplemental material S9. A few specific studies are highlighted below. A Canadian prospective population-based surveillance study found that among adults 50 years of age and older hospitalized with RSV, 13.7% required ICU admission and 6.4% required mechanical ventilation (similar to influenza) between 2012 and 2015 ((2)). As with other clinical outcomes, risk increased with age and the presence of comorbidities although data was more limited by specific age groups ((7,14)). A SR from developed countries (North America, Europe, Western Pacific) found a higher proportion of adults 18 years of age and older considered at risk of complications of infection was admitted to the ICU (26.7% vs. 5.0%), required oxygen use (23.8%–50.0% vs. 13.6%–14.8%), and was discharged to care (4.2%–17.3% vs. <1%) compared to adults 60 years of age and older ((7)). A Canadian prospective cohort study found that among adults 50 years of age and older with a history of COPD hospitalized with RSV during the winter seasons of 2011 to 2015, 17.9% required ICU admission, 9.0% were mechanically ventilated, and 23.6% needed non-invasive ventilation ((15)). A surveillance study from the US found that patients who resided in LTC or other chronic care facilities had a 4.43 (95% CI: 2.23–8.82) times higher likelihood of severe clinical outcomes (i.e., ICU admission, receiving mechanical ventilation and/or death) compared to patients with other living situations at admission ((16)).

**Canadian hospitalization data:** Across 12 seasons, among the 19,436 hospitalizations associated with RSV, 3,184 (16%) required ICU admission and among the 6,314 hospitalizations due to RSV, 642 (10%) required ICU admission. The average rate of ICU admissions associated with RSV among adults 50 years of age and older across the 12 seasons were 2.6 per 100,000 population and increased with age (1.1 in adults 50–59 years, 2.4 in 60–69 years, 4.3 in 70–79 years, and 6.0 in adults 80 years of age and older) (**Table 3**). Rates of ICU admissions due to RSV in Canadian older adults followed the same trend; however, rates were much lower.

**Table 3:** Number and rate of intensive care unit (ICU) admissions associated with and due to respiratory syncytial virus (RSV), percentage of RSV hospitalizations resulting in an ICU admission, by age group (years), seasons 2010–2011 to 2019–2020 and 2021–2022 to 2022–2023^a,b^

Age group (years)	Among hospitalizations associated with RSV^c^			Among hospitalizations due to RSV^d^		
	Number of ICU admissions	Rate per 100,000 population^e^	% of hospitalizations requiring ICU	Number of ICU admissions	Rate per 100,000 population^e^	% of hospitalizations requiring ICU
50–59	504	1.1	26	101	0.2	17
60–69	896	2.4	23	155	0.4	14
70–79	969	4.3	19	174	0.8	12
80+	815	6.0	9	212	1.5	7
Total^f^	3,184	2.6	16	642	0.5	10

Abbreviations: ICU, intensive care unit; RSV, respiratory syncytial virus

^a^ Canada, excluding Québec

^b^ Canadian Discharge Abstract Database

^c^ Hospitalizations associated with RSV were identified using ICD-10 codes J12.1, J20.5, J21.0 or B97.4 found anywhere from diagnosis 1 through 25

^d^ Hospitalizations due to RSV were identified using ICD-10 codes J12.1, J20.5, J21.0 or B97.4 recorded as diagnosis 1

^e^ Aggregated rate by age group was calculated by averaging the number of ICU by age group, by season and dividing by the average population of that age group across the study period

^f^ Total column values were calculated by aggregating ICU across all seasons in the study period. The average population of each season during the study was used to calculate rates

Regardless of the type of RSV hospitalization (associated with or due to), the number and rate of ICU admissions increased with age but the proportion of hospitalizations requiring ICU admissions decreased with age.

### Death associated with respiratory syncytial virus infection

**Rapid review:** Five SRs and eleven observational studies, including four Canadian studies, reported data on death associated with RSV infection. Full results are listed in Supplemental material S9. A few specific studies are highlighted below. Although evidence is more limited than for other clinical outcomes, in general the CFR among adults admitted to hospital is approximately 5%–10% which increases with age and the presence of one or more comorbidities. A SR of developed countries found an overall RSV-related CFR of 8.2% (95% CI: 5.5–11.9%) among adults 60 years of age and older and 9.9% (95% CI: 6.7%–14.4%) among adults 18 years of age and older considered at higher risk ((7)). Another systematic review and meta-analysis found that the in-hospital case fatality rate was higher in adults 65 years of age and older than adults 50–64 years of age ((1)). Similarly, two studies from Ontario found that among patients hospitalized with RSV, 30-day all-cause mortality rates increased with age ((12,17)). A US prospective cohort study found that the CFR was higher in adults admitted from LTC facilities (38%) than in those admitted from the community (3%, *p*<0.001) ((18)).

**Canadian hospitalization data:** Across 12 seasons, 1,668 in-hospital deaths among RSV associated hospitalizations were reported in adults 50 years of age and older, corresponding to an in-hospital CFR of 9% (**Table 4**). Among these in-hospital deaths, 397 were among those hospitalized due to RSV, corresponding to an in-hospital CFR of 6%. The average rate of in-hospital deaths associated with RSV in adults 50 years of age and older across the 12 seasons was 1.4 per 100,000 population and increased with age (the rates of in-hospital deaths in hospitalizations associated with RSV per 100,000 population by age groups were the following: 0.2 in adults 50–59 years old, 0.6 in 60–69 years, 1.7 in 70–79 years, and 6.7 in adults 80 years of age and older). The rates of in-hospital deaths among older adults hospitalized due to RSV in Canada followed the same trend; however, rates were much lower.

**Table 4:** Number and rate of in-hospital deaths associated with and due to respiratory syncytial virus, case fatality rate, by age group (years), seasons 2010–2011 to 2019–2020 and 2021–2022 to 2022–2023^a,b^

Age group (years)	Among hospitalizations associated with RSV^c^			Among hospitalizations due to RSV^d^		
	Number of in-hospital deaths	Rate per 100,000 population^e^	CFR (%)	Number of in-hospital deaths	Rate per 100,000 population^e^	CFR (%)
50–59	110	0.2	6	18	0.0	3
60–69	244	0.6	6	44	0.1	4
70–79	390	1.7	8	72	0.3	5
80+	924	6.7	11	263	1.9	8
Total^f^	1,668	1.4	9	397	0.3	6

Abbreviations: CFR, case fatality rate; RSV, respiratory syncytial virus

^a^ Canada, excluding Québec

^b^ Canadian Discharge Abstract Database

^c^ Hospitalizations associated with RSV were identified using ICD-10 codes J12.1, J20.5, J21.0 or B97.4 found anywhere from diagnosis 1 through 25

^d^ Hospitalizations due to RSV were identified using ICD-10 codes J12.1, J20.5, J21.0 or B97.4 recorded as diagnosis 1

^e^ Aggregated rate by age group was calculated by averaging the number of deaths by age group, by season and dividing by the average population of that age group across the study period

^f^ Total column values were calculated by aggregating deaths across all seasons in the study period. The average population of each season during the study was used to calculate rates

Regardless of the type of RSV death (in hospitalizations associated with or due to RSV), both the number and rates of death and CFR increased with age.

## Discussion

The rapid review offers insight into the burden of RSV disease in older adults and adults with underlying medical conditions, with a focus on high-income countries such as Canada, the US and European countries. This review is also supported with hospitalization data to further describe RSV burden of disease in Canada.

Evidence from the rapid review suggests that medically attended RSV infections in high-income countries are frequent in older adults and those with underlying medical conditions. The incidence of RSV RTI increases with age as well as the presence of comorbidities, including cardiorespiratory disease, diabetes and immunocompromising conditions. While the incidence of hospitalization varies between studies, risk of hospitalization associated with RSV increases consistently with age. Depending on age and risk factors, adults 18 years of age and older with underlying medical conditions are more likely to have a hospitalization associated with RSV infection than those without. Patients who reside in LTC or other chronic care facilities have a higher likelihood of severe clinical outcomes compared to patients with other living situations upon hospital admission. Moreover, ICU admission associated with RSV increases with age and presence of comorbidities, with approximately 10% of older hospitalized older adults requiring ICU admission. There were more limited data on deaths associated with RSV. The CFR among those admitted to hospital varied between studies but is approximately 5%–10% and increases with age.

Canadian administrative hospitalization data generally support the findings of the rapid review. Over 12 respiratory seasons between August 2010 and September 2023, it was found that RSV-associated hospitalization rates increased with age and that finding was consistent for each season. The average rates of hospitalization associated with RSV in adults 50 years of age and older was estimated at 16 per 100,000 population. Overall, 16% of hospitalizations associated with RSV resulted in an ICU admission corresponding to an average rate of 2.6 per 100,000 population for adults 50 years of age and older. Rate of hospitalization associated with RSV resulting in ICU admissions increased with age; however, the proportion of hospitalizations requiring ICU admission decreased with age. The average CFR among adults 50 years of age and older was 9% and in-hospital death among hospitalizations associated with RSV increased with age. Hospitalizations, ICU admissions and deaths due to RSV followed the same trend; however, calculated values were lower than those associated with RSV.

Although there is general alignment between the rapid review and Canadian hospitalization data analysis, with increasing risk with age and specific conditions, some differences can be noted. Findings from the Canadian hospitalization data were usually lower than what is reported in the literature. Discrepancies can be explained by differences in methodology used between studies. Individual study characteristics such as study population, case definitions, study period, and data source can lead to discrepancies between the observed incidence rates. Few studies reported data on Canadian adults and heterogeneity between study results limits the generalizability of the findings. Another limitation of the rapid review is the inclusion of RSV infection not limited to laboratory confirmed infection potentially leading to an overestimation of RSV incidence.

Currently, Canada has limited enhanced national RSV surveillance data and leveraging administrative health data from CIHI DAD helped address those evidence gaps to supplement the evidence on RSV burden of disease in adults to inform the development of immunization recommendation. However, known limitations of healthcare administrative data are expected to lead to underestimation of RSV incidence especially due to limits in viral identification and undertesting in patients ((19)). Of note, rates from CIHI DAD were higher in the more recent period, which could be partly due to more frequent testing. Other limitations of the Canadian hospitalization data include the exclusion of Québec data, a large Canadian province, differing coding practices between hospitals and changes in testing and admission practices during the study period, especially in respiratory season following the COVID-19 pandemic.

The descriptive analyses provided information on general trends of severe outcomes of RSV RTI in older adults (19). Although the rapid review and healthcare administrative data analysis methodologies each have their drawbacks, the combination of these analyses provides an interdisciplinary view of the burden of RSV in older adults to support vaccine program decision-making. Enhanced national surveillance programs for RSV are in development where timely data variables of interest can be collected specifically for surveillance activities and to support policy and decision-making. These analyses may be revisited as additional data becomes available from the literature or from the Canadian surveillance landscape.

## Appendix

Supplemental material is available upon request to the author: elissa.abrams@phac-aspc.gc.ca
